# Particulate Air Pollution Exposure and Expression of Viral and Human MicroRNAs in Blood: The Beijing Truck Driver Air Pollution Study

**DOI:** 10.1289/ehp.1408519

**Published:** 2015-06-12

**Authors:** Lifang Hou, Jitendra Barupal, Wei Zhang, Yinan Zheng, Lei Liu, Xiao Zhang, Chang Dou, John P. McCracken, Anaité Díaz, Valeria Motta, Marco Sanchez-Guerra, Katherine Rose Wolf, Pier Alberto Bertazzi, Joel D. Schwartz, Sheng Wang, Andrea A. Baccarelli

**Affiliations:** 1 Department of Preventive Medicine, and; 2 Robert H. Lurie Comprehensive Cancer Center, Feinberg School of Medicine, Northwestern University, Chicago, Illinois, USA; 3 Department of Environmental Health, Harvard T.H. Chan School of Public Health, Boston, Massachusetts, USA; 4 Bioinformatics Infrastructure Facility, University of Rajasthan, Jaipur and Barupal Research Foundation, Jaisalmer, India; 5 Department of Pediatrics, University of Illinois, Chicago, Illinois, USA; 6 Institute for Public Health and Medicine, Feinberg School of Medicine, Northwestern University, Chicago, Illinois, USA; 7 Department of Safety Engineering, China Institute of Industrial Health, Beijing, China; 8 Center for Health Studies, Universidad del Valle de Guatemala, Guatemala City, Guatemala; 9 Center of Molecular and Genetic Epidemiology, Department of Environmental and Occupational Health, University of Milan and IRCCS Maggiore Policlinico Hospital, Mangiagalli and Regina Elena Foundation, Milan, Italy; 10 Department of Occupational and Environmental Health, Peking University Health Science Center, Beijing, China

## Abstract

**Background:**

MicroRNAs (miRNAs) are post-transcriptional gene suppressors and potential mediators of environmental effects. In addition to human miRNAs, viral miRNAs expressed from latent viral sequences are detectable in human cells.

**Objective:**

In a highly exposed population in Beijing, China, we evaluated the associations of particulate air pollution exposure on blood miRNA profiles.

**Methods:**

The Beijing Truck Driver Air Pollution Study (BTDAS) included 60 truck drivers and 60 office workers. We investigated associations of short-term air pollution exposure, using measures of personal PM_2.5_ (particulate matter ≤ 2.5 μm) and elemental carbon (EC), and ambient PM_10_ (≤ 10 μm), with blood NanoString nCounter miRNA profiles at two exams separated by 1–2 weeks.

**Results:**

No miRNA was significantly associated with personal PM_2.5_ at a false discovery rate (FDR) of 20%. Short-term ambient PM_10_ was associated with the expression of 12 miRNAs in office workers only (FDR < 20%). Short-term EC was associated with differential expression of 46 human and 7 viral miRNAs, the latter including 3 and 4 viral miRNAs in office workers and truck drivers, respectively. EC-associated miRNAs differed between office workers and truck drivers with significant effect modification by occupational group. Functional interaction network analysis suggested enriched cellular proliferation/differentiation pathways in truck drivers and proinflammation pathways in office workers.

**Conclusions:**

Short-term EC exposure was associated with the expression of human and viral miRNAs that may influence immune responses and other biological pathways. Associations between EC exposure and viral miRNA expression suggest that latent viral miRNAs are potential mediators of air pollution–associated health effects. PM_2.5_/PM_10_ exposures showed no consistent relationships with miRNA expression.

**Citation:**

Hou L, Barupal J, Zhang W, Zheng Y, Liu L, Zhang X, Dou C, McCracken JP, Díaz A, Motta V, Sanchez-Guerra M, Wolf KR, Bertazzi PA, Schwartz JD, Wang S, Baccarelli AA. 2016. Particulate air pollution exposure and expression of viral and human microRNAs in blood: the Beijing Truck Driver Air Pollution Study. Environ Health Perspect 124:344–350; http://dx.doi.org/10.1289/ehp.1408519

## Introduction

Air pollution is a major public health concern in the United States and worldwide, accounting for approximately 3.7 million premature deaths in 2012 [World Health Organization (WHO) 2014]. Epidemiological studies have demonstrated that peaks in ambient levels of air particulate matter (PM) pollution, of which traffic is a major source, are rapidly followed, within hours or days after exposure, by increased hospitalization and death, particularly from cardiovascular disease (CVD) (Brook et al. 2010; Wang et al. 2013; Wong et al. 2010) and pulmonary disorders (Laumbach and Kipen 2012). In the presence of stagnating weather, peaks more than 10 times higher than background PM_2.5_ (particulate matter with an aerodynamic diameter ≤ 2.5 μm) levels are still frequently recorded in U.S. cities (Bellavia et al. 2013). Inhaled traffic-derived PM up-regulates blood leukocyte pro-inflammatory responses and oxidative stress, two major cardiopulmonary pathways. In blood samples of healthy volunteers collected before, during, and after the 2008 Olympics in Beijing, China, changes in air pollution levels across the different periods were found to be associated with differences in several inflammation biomarkers, such as C-reactive protein (CRP) and fibrinogen (Rich et al. 2012). Blood leukocyte gene expression profiling in 18 PM-exposed welders compared with 10 unexposed controls suggested that associations between PM and systemic inflammatory responses may be initiated through changes in gene expression (Wang et al. 2005).

MicroRNAs (miRNAs) are small (20–24 nucleotides) noncoding RNAs that regulate gene expression by inducing mRNA cleavage or reducing translation (Baccarelli and Bollati 2009). Inflammation and oxidative stress have been shown to affect miRNA expression in blood leukocytes (Xiao and Rajewsky 2009). Recent *in vitro* studies have shown that diesel exhaust particles (DEPs), a primary component of PM from vehicular traffic, affect the expression of specific miRNAs (Jardim et al. 2009; Yamamoto et al. 2013). In addition to human miRNAs, miRNAs of viral origin are detectable in human cells (Grundhoff and Sullivan 2011). Expression of viral miRNAs in human cells may result from active infections or, more often, from the expression of latent retroviral sequences integrated in the host DNA (Grundhoff and Sullivan 2011). MiRNA profiles, particularly viral miRNA profiles, are still understudied in relation to traffic pollution in real-life urban conditions.

In the present study, we investigated two highly exposed groups of 60 truck drivers and 60 office workers in Beijing to examine whether exposure to air pollution, traced through PM_2.5_, PM_10_ (≤ 10 μm), and traffic elemental carbon (EC) measures, is associated with miRNA profiles in blood leukocytes. We used the NanoString nCounter miRNA Analysis system, a novel digital technology that can accurately distinguish between highly similar miRNAs with high specificity (Knutsen et al. 2013). Traffic-derived PM and its health implications are a major concern in Beijing because of its high population density and rapid increases in vehicular traffic. The two study groups both had high exposure levels and were selected to sample on different types of exposures: Truck drivers are directly exposed to traffic emissions, particularly from diesel exhausts and road dusts; office workers represented the highly exposed urban residential population of Beijing, which has a higher exposure to secondary oxidized traffic particles than truck drivers (Baccarelli et al. 2014; Hou et al. 2014). To identify specific short-term changes in miRNA, we studied each participant on 2 different examination days 1–2 weeks apart and assessed each participant’s exposure on each examination day using a personal monitor.

## Materials and Methods

*Study participants*. The Beijing Truck Driver Air Pollution Study (BTDAS), conducted between 15 June and 27 July 2008, included two groups with high exposure to air pollution: 60 truck drivers and 60 office workers (Baccarelli et al. 2011). We examined each participant on 2 separate days separated by a 1- to 2-week interval. A self-administered questionnaire was used to collect detailed information on demographics, lifestyle, and other exposures. Information on time-varying factors, for example, smoking, was obtained both for past lifestyle patterns and for consumption on the examination day. Individual written informed consent was provided by the participants and institutional review board approval at all participating institutions was obtained before study participant recruitment.

*Ambient PM_10_, personal PM_2.5_, and personal EC measurements*. We used daily averages of ambient PM_10_ computed from data obtained from 27 monitoring stations distributed representatively across the city to estimate the average PM_10_ level in Beijing. We measured PM_2.5_ and EC on both examination days using small-weight gravimetric samplers worn by the study participants during the 8 hr of work as previously described (Baccarelli et al. 2011). To estimate EC, the blackness of the same filters used to measure PM_2.5_ was assessed using an EEL Model M43D smoke stain reflectometer (Diffusion Systems Ltd., London, UK), applying the standard black-smoke index calculations of the absorption coefficients based on reflectance (International Organization for Standardization 1993). Furthermore, to better characterize the sources of air pollution exposures in truck drivers and office workers, we measured elemental components of PM_2.5_ using an XRF PANanalytical Epsilon 5 analyzer (Almelo, the Netherlands). For each element, we calculated enrichment factors (EF) for truck drivers (TD) relative to office workers (OW) as follows: EF(x)t = [x_(TD)_/PM_2.5(TD)_]/[x_(OW)_/PM_2.5(OW)_], where [x_(TD)_/ PM_2.5(TD)_] is the ratio of the mean concentration of element x over the mean PM_2.5_ mass in truck drivers, and [x_(OW)_/PM_2.5(OW)_] is the ratio of the mean concentration of the same element x over the mean PM_2.5_ mass in office workers. As shown in Supplemental Material, Figure S1, truck drivers were more likely to be exposed to PM_2.5_ enriched in components typically found in vehicle exhausts and road dust, such as calcium (Ca), silicon (Si), and chlorine (Cl).

*NanoString nCounter assay for miRNA profiling*. A total of 240 peripheral blood samples from 120 participants was collected in PAXgene Blood RNA Tubes (Qiagen, Valencia, CA) at the end of each study day (between 1600 and 1800 hours). Total RNA was extracted using the PAXgene Blood-RNA Kit Qiagen-763134 (Qiagen). All samples had optical density ratios 280/260 ≥ 1.9 and 260/230 ≥ 1.8. RIN (RNA integrity number) scores showed excellent RNA quality (mean ± SD, 8.3 ± 0.9). We profiled miRNAs using NanoString nCounter-miRNA expression analysis (NanoString Technologies, Seattle, WA). This method measures 654 endogenous human-associated miRNAs and 80 viral miRNAs expressed in human cells. Each of the total 21 plates that accommodated the 252 study samples (240 samples plus 12 replicates) included 6 samples from office workers and 6 from truck drivers, which were randomized by group labels. Raw data were processed using the NanoStringNorm R package (Waggott et al. 2012). The raw data were log_2_ transformed and normalized using the mean of the 6 positive controls, which were used to calculate a scaling factor in each column (lane/sample) as suggested by NanoString. The internal positive spike controls were present in each reaction to account for minor differences in hybridization, purification, or binding efficiencies. The data were further background corrected by subtracting the mean of the 6 negative controls followed by quantile normalization. Only miRNAs with detectable values in more than 90% of all samples were retained for downstream analyses. Principal components analysis (PCA) was performed on the processed data to identify possible outlier samples. The nCounter miRNA data were also confirmed through cross-platform validation in 20 randomly selected study samples using the TaqMan OpenArray Real-Time PCR Plates (Life Technologies, Carlsbad, CA) on the QuantStudio 12K Flex Real-Time PCR System. The average Pearson correlation coefficient was 0.73 (0.63–0.79) between the two platforms, thus confirming the robustness of the nCounter platform (see Supplemental Material, Figure S2).

The raw and processed miRNA data have been deposited into the NCBI (National Center for Biotechnology Information) Gene Expression Omnibus (accession number GSE63087).

*Statistical analysis*. Separate mixed-effects regression models were used to estimate associations of within- and between-subject variation in exposure to PM_2.5_, EC, and PM_10_ with miRNA expression among all participants combined (pooled analysis), and in truck drivers and office workers separately (SAS 9.1, PROC MIXED; SAS Institute Inc., Cary, NC). We examined within-subject effects representing variation in miRNA expression due to variation in exposure with time, as well as between-subject effects representing variation in miRNA expression due to individual differences in exposures. We fitted models adjusted for variables either not matched or incompletely matched by design between the two groups, that is, age, sex, body mass index (BMI) (continuous), smoking status, number of cigarettes smoked on the examination day (continuous), examination day, work hours per week, outdoor temperature (continuous), and dew point (continuous). The mixed-effects model was

γ*_ij_* = β_0_ + β_mean_ (Exposure_mean_) + β_diff_ (Exposure_diff_) + β_1_X_1_*_ij_* + … + β*_k_*X*_kij_* + ξ*_i_* + e*_ij_*, [1]

where γ*_ij_* is the mean expression of a miRNA investigated for the *i*th subject on the *j*th examination day (*i* = 1,…,n; *j* = 1,2); β_0_ is the overall intercept; β_mean_ is the regression coefficient for Exposure_mean_, i.e., the mean exposure on both work days (between-subject effect); β_diff_ is the regression coefficient for the difference between Exposure_diff_, that is, the difference between exposures on a single work day and mean exposure on both work days (within-subject effect); β_2_…β*_k_* are the regression coefficients for the covariates, which included age, sex, BMI, smoking status, number of cigarettes smoked on the examination day, examination date, work hours per day, and outdoor temperature and dew point on the examination day; ξ*_i_* is the random effect for the subject; and e*_ij_* is the residual error term. All tests were two-sided, and miRNA expression changes with a Benjamini–Hochberg false discovery rate (FDR) (Benjamini and Hochberg 1995) < 20%, following previous miRNA expression association studies (Barry et al. 2012; Dorval et al. 2014; Gowrishankar et al. 2014), were considered significant and selected for pathway analysis.

*Bioinformatics analysis*. A computationally predicated database, miRBase (Griffiths-Jones et al. 2008), was used as the primary source for gene targets of differentially expressed miRNAs. The Miranda algorithm used in miRBase assigns *p-*values to individual miRNA-3′-UTR target binding sites for assessing the statistical significance of predicated complementarity between miRNA and target. Because a gene can have binding sites with different *p-*values, we used two *a priori* stringent cutoff *p*-values (*p <* 10^–6^ and *p* < 10^–8^) that can provide robust targets for a miRNA. For more comprehensive and reliable target identification, the annotation was further complemented by two experimental databases, miRTarBase (Hsu et al. 2011) and TarBase (Sethupathy et al. 2006).

KEGG (Kyoto Encyclopedia of Genes and Genomes) (Kanehisa et al. 2002) pathway and GO (Gene Ontology) (Gene Ontology Consortium 2008) analyses for pathway and biological process enrichment were performed using the combined target genes of all miRNAs that were significantly associated with EC (FDR < 20%) in each group. To consider enrichment significant, a threshold of Benjamini–Hochberg FDR < 5% for a KEGG pathway and 1% for GO terms were set with minimum eight genes in a gene set. We created functional interaction (FI) networks using target genes selected with miRBase (cutoff *p <* 10^–8^), TarBase, and miRTarBase (see Supplemental Material, Figure S3) for each study group. We set the node size according to the node degree to highlight the most-connected genes. We performed pathway enrichment analysis of the genes in the FI networks using the Reactome FI Cytoscape plugin (version 8.2.1), which can be used to construct the networks based on information from both predicated and physical interaction among genes (Wu and Stein 2012). A network-clustering algorithm (Newman 2006) provided by the Reactome FI plugin was used for module identification with a minimum of seven nodes.

## Results

*Characteristics of study participants and air pollution exposure measurements*. The characteristics and personal PM_2.5_, personal EC, and ambient PM_10_ exposure levels of the 60 office workers and 60 truck drivers are shown in Table 1. Briefly, truck drivers were older, had higher BMI, higher personal levels of PM_2.5_ and EC, smoked more cigarettes during the examination period, and had longer working hours than office workers did.

**Table 1:** Characteristics and exposure levels of the study participants [mean ± SD or *n* (%)].

Characteristic	Office workers (*n* = 60)	Truck drivers (*n* = 60)	*p*‑Value^*a*^
Personal PM_2.5_ level (μg/m^3^)	94.6 ± 64.9	126.8 ± 68.8	< 0.001
Personal EC level (μg/m^3^)	13.1 ± 4.0	17.3 ± 6.7	< 0.001
Ambient PM_10_ level (μg/m^3^)	116.7 ± 50.2	123.5 ± 50.1	0.29
Age (years)	30.3 ± 8.0	33.5 ± 5.7	0.01
Sex
Male	40 (66.7)	40 (66.7)
Female	20 (33.3)	20 (33.3)	0.99
BMI (kg/m^2^)	22.8 ± 3.4	24.3 ± 3.2	0.01
Smoking status
Never smoker	35 (58.4)	34 (56.7)
Former	2 (3.3)	2 (3.3)
Current	23 (38.3)	24 (40.0)	0.99
Cigarettes smoked during examination time^*b*^	2.6 ± 5.2	6.4 ± 9.4	< 0.001
Work hours per week	50.6 ± 11.0	67.3 ± 14.0	< 0.001
Average temperature (°C)^*c*^	25.4 ± 2.5	25.3 ± 2.5	0.96
Average dew point (°C)^*c*^	20.6 ± 2.1	20.6 ± 2.1	0.93
^***a***^*p*‑Values were calculated using Student’s *t*-test and Fisher’s exact test for continuous and categorical variables, respectively. *p*-Values were obtained from mixed-effects regression models. ^***b***^Only current or former smokers. ^***c***^Cumulative of the 2 study days. Based on 240 total observations (120 study days for office workers and 120 study days for truck drivers).			

*MiRNA expression data*. We obtained high correlation of miRNA expression measures among 12 technical replicates using processed data (*r >* 0.96). After background correction and normalization, detectable miRNAs in > 90% of the 240 total samples were included for further analysis. As a result, 166 miRNAs remained in the analysis, including 159 human and 7 viral miRNAs. This number is consistent with the expected detection rate, as indicated in the NanoString nCounter documentation for the miRNA platform (NanoString Technologies 2013). We dropped 12 samples considered obvious outliers due to low RNA yields based on the PCA plot (see Supplemental Material, Figure S4). All 12 samples were from 12 different participants; these include 8 samples from office workers and 4 samples from truck drivers. The technical robustness of the NanoString platform was demonstrated by showing that there was no difference in the expression value of these positive controls across the 21 plates (*p* > 0.995, one-way analysis of variance) (see Supplemental Material, Figure S5). Negligible plate effect on miRNA expressions was seen in the PCA plot (see Supplemental Material, Figure S6). The mean correlation (Pearson’s *r*) of miRNA expression among paired days was 0.860 for office workers and 0.862 for truck drivers.

**Figure 1:**
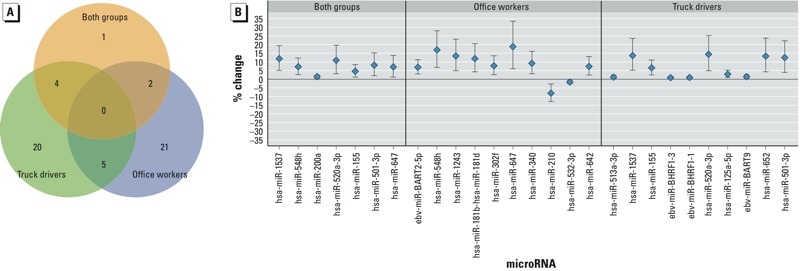
MiRNAs associated with short-term EC level. (*A*) Venn diagram showing the number of miRNAs associated with EC level in each analysis group (office workers, truck drivers, and the combined samples). (*B*) Differentially expressed miRNAs ordered by *p*-value at FDR < 20% in the combined samples, and top 10 significant miRNAs in office workers, and in truck drivers. Percent change in miRNA expression is shown on the *y*-axis. Up-regulated miRNAs were above the zero percent change value, and down-regulated miRNAs were below the zero percent change value. 95% CIs are represented by error bars.

*MiRNA signatures associated with air pollution exposure*. Short-term EC average level was associated with differential expression of 46 human miRNAs and all 7 viral miRNAs detected in the blood samples (FDR < 20%) using the mixed-effects model. Only 7 miRNAs were significantly associated with EC in the pooled analysis. In contrast, when stratified on occupational group, 28 miRNAs were significant for office workers, and 29 for truck drivers, with only 5 miRNAs being common to both groups (hsa-miR-125a-5p, hsa-miR-1274a, hsa-miR-600, hsa-miR-1283, and hsa-miR-10a) and none being common to all three groups (Figure 1A).

Among the significant miRNAs with FDR < 20% in each occupational group, EC was associated with 3 viral miRNAs (EBV-miR-BART2-5p, EBV-miR-BART6-5p, and KSHV-miR-K12-9) in office workers and 4 viral miRNAs (EBV-miR-BHRF-1-3, EBV-miR-BHRF-1-1, EBV-BART-9, and HIV1-miR-H1) in truck drivers (see Supplemental Material, Table S1). All 7 miRNAs in pooled analysis, and the top 10 significant miRNAs based on *p*-values in each groups are shown in Figure 1B. From the same mixed-effects model, we observed predominant up-regulation of miRNA in truck drivers (28 up-regulated vs. 1 down-regulated) and both up- and down-regulation in office workers (16 up-regulated vs. 12 down-regulated) (see Supplemental Material, Table S1). In a pooled analysis that combined data from both groups, 7 miRNAs (all up-regulated) were associated with EC at FDR < 20% (Figure 1B; see also Supplemental Material, Table S1). For highly significant miRNAs selected at FDR < 10% by group analysis, we observed significant interactions (*p*-value < 0.05) between exposure and occupational groups (see Supplemental Material, Table S2). In a sensitivity analysis excluding current/former smokers (25 participants from office workers and 26 participants from truck drivers), we identified 12 miRNAs in office workers and 5 miRNAs in truck drivers (FDR < 20%) (see Supplemental Material, Table S3). Specifically, 10 miRNAs in office workers (including 2 viral miRNAs) and 2 miRNAs in truck drivers (both viral miRNAs) were observed in both analyses using all participants or only nonsmokers, and all overlapped miRNAs showed expression change in the same direction. These results indicated that the significance of the association of certain miRNAs with EC was not affected by smoking, despite the fact that the sample size was limited in the sensitivity analysis. We compared the miRNAs correlated with EC and other exposure measurements (PM_2.5_ and PM_10_) and found that PM_10_ had short-term (within-subject) effects on the expression of 12 miRNAs in office workers and one miRNA in the pooled analysis at FDR < 20% (see Supplemental Material, Table S4). In truck drivers, we observed no significant miRNAs associated with PM_10_. Personal exposure to PM_2.5_ was not associated with any miRNAs at FDR < 20% (see Supplemental Material, Table S5).

*Function and pathway analysis of miRNA-targeted genes*. We identified the target mRNAs of the miRNAs associated with short-term changes in EC levels using multiple databases. We used miRBase to identify the target mRNAs, complemented by miRTarBase and TarBase. Using a threshold of *p <* 10^–6^, we identified 218 target mRNAs in office workers, 595 target mRNAs in truck drivers, and 219 target mRNAs in the pooled analysis. In contrast, at *p <* 10^–8^, we identified 168 target mRNAs in office workers, 503 target mRNAs in truck drivers, and 187 target mRNAs in the pooled analysis. Only 8% (61) were common between office workers and truck drivers at *p <* 10^–6^, and 7% (44) at *p <* 10^–8^. Ten mRNA targets were common between all three analyses at *p<* 10^–6^, and 9 mRNA targets were common between the three analyses at *p <* 10^–8^.

**Figure 2:**
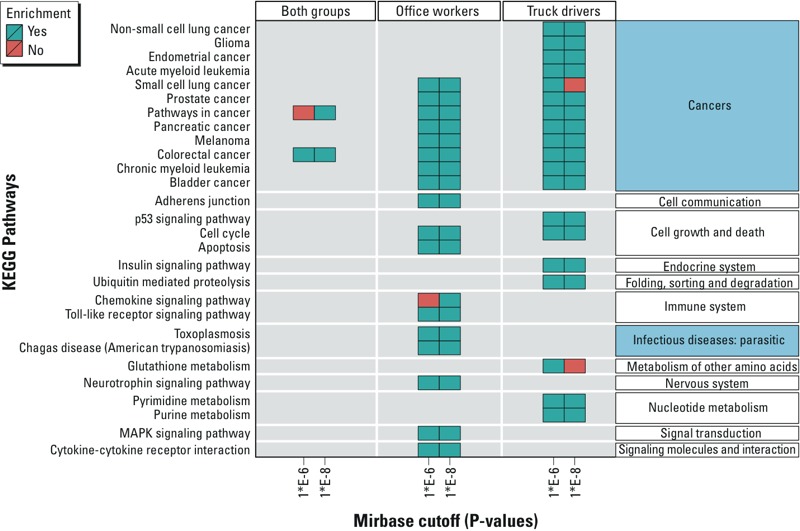
Pathway analysis. KEGG pathways enriched for gene targets of miRNAs associated with short-term EC level analyzed by Fisher’s exact test at FDR < 5%. Two threshold cutoffs (10^–6^ and 10^–8^) for gene selection in the miRBase database are shown on the *x*-axis. Color of a tile represents enrichment of a pathway: green = yes, red = no. Higher-order KEGG categories are shown on the right side. Higher-order categories that include human disease pathways are shown in blue. The KEGG analysis showed enrichment of 28 different pathways (14 non-disease pathways and 14 disease pathways) at either miRBase threshold. Results from pathway analysis were robust to the threshold cutoffs in miRBase for the identification of mRNA targets.

We conducted pathway analysis of the gene targets identified in miRBase at either *p*-value threshold. The pathway analysis showed enrichment of 28 canonical pathways from the KEGG database (14 non-disease pathways and 14 disease pathways) at either miRBase threshold (Figure 2; see also Supplemental Material, Table S6). Notably, two pathways (“colorectal cancer” and “pathways in cancer”) were enriched in the pooled analysis of both groups, as well as in office workers and truck drivers separately. Nine pathways (8 cancer pathways and 1 cell cycle pathway) were found to be enriched in both office workers and truck drivers. The non-disease pathways for office workers were related to the immune response, cellular communication and cell growth, while those for truck drivers were largely linked with nucleotide and amino acid metabolic activities (Figure 2; see also Supplemental Material, Table S6). As shown in Figure 2 (see also Supplemental Material, Figure S7), results from the KEGG pathway analysis were robust in terms of the threshold used in miRBase for the identification of mRNA targets. Of the 18 total pathways identified among office workers, 17 were shared using both thresholds. Of the 19 pathways identified among truck drivers, 17 were shared using both thresholds (see Supplemental Material, Figure S7).

We also conducted functional analysis based on the GO terms (see Supplemental Material, Figure S8 and Table S7). At any miRbase cutoff, the GO analysis showed that the highest proportion of biological processes was related to biological regulation (three of total five GO terms related to transcription regulation) in the pooled analysis, immune response (> 35%) in office workers (e.g., Toll-like receptor signaling–related pathways, response to drug, double-stranded DNA break), and cell growth/death (50%) in truck drivers (e.g., negative regulation of cell cycle, insulin receptor–signaling pathways, cell cycle arrest, G1 phase mitotic cell cycle, negative regulation of apoptosis process). Biological processes shared by both groups included pathway related to cell growth/death, biological regulation and metabolism, negative regulation of apoptotic process, negative regulation of cell growth, cell cycle arrest, positive regulation of transcription from RNA polymerase II promoter, negative regulation of transcription from RNA polymerase II promote, and positive regulation of transcription, DNA-dependent. Only one immune or stress response–related GO terms was enriched in truck drivers (response to drug). In the combined analysis, no GO term was robust in terms of the miRBase cutoff. In office workers, 23 of the 31 GO terms were found at both thresholds. In truck drivers, 13 of the 20 GO terms were found at both thresholds (see Supplemental Material, Figure S7).

In contrast, pathways and functional analysis for miRNAs associated with PM_10_ and PM_2.5_ showed that one KEGG pathway (“pathways in cancer”) and three GO terms (“positive regulation of transcription from RNA polymerase II promoter,” “negative regulation of transcription from RNA polymerase II promoter,” and “negative regulation of cell proliferation”) were enriched in short-term effect of PM_10_ in office workers (data not shown). For the other analyses on truck drivers and two groups combined in relation to PM_10_ and PM_2.5_, no significantly enriched KEGG pathways or GO terms associated with PM_10_ or PM_2.5_ were found (data not shown).

**Figure 3:**
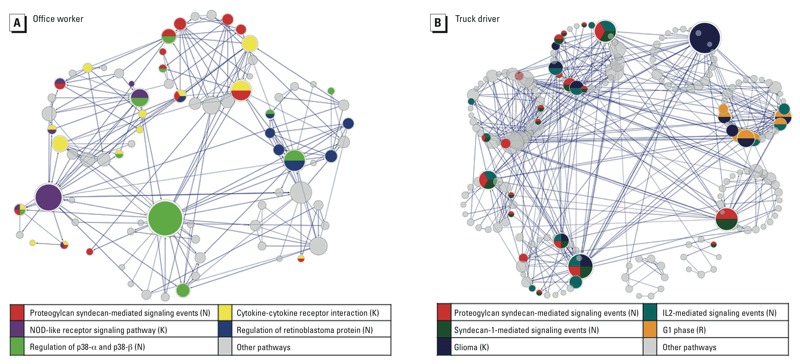
Reactome functional interaction (FI) networks. Reactome FI networks showed interactions of the target mRNAs based on physical or predicated connections. Abbreviations: K, KEGG; N, National Cancer Institute; R, Reactome Database. Circles represent nodes, and circle size represents node degree (the number of connections one node has). Lines with arrows represent physical interactions between nodes. Each node color represents an enriched pathway, and multi-colored nodes represent genes enriched in multiple pathways. Modules are clusters of nodes with a minimum of seven genes. The top five significant pathways in office workers and truck drivers are listed. The network clustering algorithm showed six modules in office workers and nine modules in truck drivers.

*Pathways and modules in FI networks*. FI networks (Figure 3) linked 43% of the target mRNAs in office workers and 45% of the target mRNAs in truck drivers by their functional interactions. Pathway enrichment showed 24 pathways in office workers and 22 pathways in truck drivers. The top 5 pathways were selected based on FDR and visualized in FI networks. Only 1 of the top 5 pathways was shared between the two groups. In office workers, the two highest-degree nodes showed gene enrichment in regulation of the P38 alpha and beta pathways and the NOD-like receptor signaling pathway. In truck drivers, the highest-degree node was enriched with genes related to interleukin-2 and signaling pathways. The network-clustering algorithm showed six modules in office workers and nine modules in truck drivers.

## Discussion

In the present study, short-term air pollution exposure from traffic particles, as traced by EC levels, was associated with differential expression of 46 human and 7 viral miRNAs, but no consistent association of PM_2.5_ and PM_10_ exposures with miRNA expressions was observed among three analyses. EC-associated miRNA profiles differed between office workers and truck drivers. Significant miRNAs detected in the stratified analyses by group were masked in the pooled analysis of both groups combined. Among the significant miRNAs (FDR < 20%) in each group, EC levels were associated with 3 viral miRNAs (EBV-miR-BART2-5p, EBV-miR-BART6-5p, and KSHV-miR-K12-9) in office workers and 4 viral miRNAs (EBV-miR-BHRF-1-3, EBV-miR-BHRF-1-1, EBV-BART-9, and HIV1-miR-H1) in truck drivers. Among human miRNAs, gene function and pathway analyses demonstrated that several of the putative mRNAs targeted by the differentially expressed miRNAs are implicated in inflammation in office workers, and in cellular proliferation- and differentiation-related pathways in truck drivers.

Previous experimental (Jardim et al. 2009) and human studies (Bollati et al. 2010; Motta et al. 2013; Yamamoto et al. 2013) have shown miRNA alterations in response to metal-rich PM, PM_2.5_, or DEPs. For example, Yamamoto et al. (2013) showed altered blood expression of 15 miRNAs in response to DEP exposure in asthma patients, including 4 miRNAs identified in office workers (hsa-miR-142-3p, hsa-miR-21, hsa-miR-125a-5p, and hsa-miR-937) and 2 miRNAs identified in truck drivers (hsa-miR-125a-5p and hsa-miR-320a) in our study. Our finding on miR-146a in office workers is consistent with data from our previous study of healthy Italian steel workers (Bollati et al. 2010; Motta et al. 2013). However, we also found results that were at variance with previous studies. For instance, in office workers we observed up-regulation of miR-937 in relation to EC level, whereas Yamamoto et al. (2013) reported down-regulation of miR-937 in relation to DEP exposure.

The five overlapping miRNAs found in both office workers and truck drivers were all down-regulated. These miRNAs have been reported to participate in cell cycle, proliferation, and apoptosis processes. For example, miR-125a has been shown to target the ErbB pathway in acute myeloid leukemia cells (Ufkin et al. 2014), and miR-10a has been linked to cell proliferation control via targeting of the PI3 kinase pathway (Hu et al. 2014). The combined analysis and the interaction analysis showed effect modifications by group. Truck drivers and office workers have substantially different distributions of changes in personal EC level between the 2 study days. This difference could have masked the effects in the pooled analysis.

The pathways associated with EC exposures differed between office workers and truck drivers. These discrepancies might originate, at least in part, from miRNA responses specific to different types of exposures or variation in exposure levels, or sample size between the two groups. Our analysis of elemental components showed that, compared with office workers, truck drivers were exposed to PM_2.5_ that was enriched in elements found in road dust. Previous studies suggest that differences in the type of exposure might differentially affect molecular mechanisms; for instance, Hoogendoorn et al. (2012) examined the effect of PM size on RNA and protein expression in normal human-derived tracheobronchial epithelial cells and found that different size ranges of PM have variation in number and in the magnitude of expression change in associated genes. Hoogendoorn et al. (2012) also detected PM effects on the expression of some of the miRNA target genes observed in our study, such as the proliferation- and carcinogen-related genes *E2F1, EGR1* targeted by hsa-miR-21 and hsa-miR-192, and the inflammation-related gene *NFKB1* targeted by miR-146a. These findings suggest that physicochemical properties of air particles may differentially affect miRNA expression.

The most novel finding in the present study was the identification of 7 viral miRNAs associated with EC levels, including 5 Epstein–Barr virus (EBV) miRNAs, 1 Kaposi’s sarcoma–associated herpes virus (KSHV) miRNA, and 1 human immunodeficiency virus-like (HIV) miRNA. More than 400 viral miRNAs have been discovered from both DNA and RNA viruses, and some of these viral miRNAs are detectable in profiles of RNA extracted from human cells (Grundhoff and Sullivan 2011). The best-characterized viral miRNAs are those coded by EBV, KSHV, and the human cytomegalovirus (HCMV) (Cullen 2006). Latent viruses may regulate host cell miRNAs or encode their own miRNAs using host cell machinery (Kincaid and Sullivan 2012). For instance, EBV can induce expression of miR-155 (Cameron et al. 2008) and miR-146a (Feederle et al. 2011) to mediate B-cell proliferation and inflammation. Virus-encoded miRNAs can target expression of key genes in the cellular pathways of the host (Ramalingam et al. 2012). KSHV-encoded miRNAs can modulate the activity of suppressor proteins involved in leukocyte recruitment (i.e., *ICAM-1*) and up-regulate the antioxidant and angiogenesis gene *HMOX1* (Gallaher et al. 2013). Although studies on the effects of environmental pollutants on miRNAs are accumulating (Bollati et al. 2010; Jardim et al. 2009; Motta et al. 2013), evidence on the effects of air pollution on viral miRNAs has been very limited. The only previous report of effects on a viral miRNA is a nonsignificant up-regulation of EBV-miR-BHRF-1 (FDR = 23%) in the previous study of DEP exposure (Yamamoto et al. 2013). Taken together, these findings may indicate a novel mechanism through which exposure-activated latent viral miRNA may exacerbate cellular effects by suppressing apoptosis, inducing cellular proliferation, and increasing inflammation. Recently, Li et al (2011) observed altered expression in a viral miRNA coded by HCMV expression in blood cells of subjects with hypertension compared to healthy controls, thus suggesting further potential links to human chronic diseases.

Our study has several notable strengths. We conducted a technical validation of personal PM_2.5_ measures that showed high reproducibility (*r* = 0.944) for our measurements. All participants were evaluated using standardized protocols for blood collection and storage. Blood RNA samples were randomized across plates to limit potential bias from plate effects, and laboratory personnel were blinded to the exposure groups and the study days. We also recognize that our study is subject to a number of limitations. Although we used multivariable models to control potential confounders, we may not be able to exclude residual confounding from unmeasured variables. We used two different thresholds to estimate the robustness of the identifications of target mRNAs in miRBase, which resulted in a number of different results dependent on the threshold used. However, in the KEGG pathway analysis, we found several pathways that were robust to the threshold specifications. The RNA isolated in our study from whole blood is derived from a mixture of cellular and extracellular miRNAs, such as miRNAs from non-leukocytes (i.e., red blood cells) and free miRNAs, which may affect our results. This study is also limited by the lack of validation of key findings in other populations.

In summary, short-term exposure to traffic-derived air particles, as reflected in EC levels, was associated with altered expression of human and viral miRNAs, which may ultimately disturb immune functions and other cellular pathways. These associations were significant only for EC, and profiles differed between office workers and truck drivers. Analyses of personal PM_2.5_ and ambient PM_10_, as well as pooled analysis of EC in both occupational groups combined, showed predominantly null results. Our finding on viral miRNAs suggests a possible mechanism through which latent viral miRNAs may exacerbate the adverse cellular effects of traffic air pollution and ultimately contribute to air pollution–associated health effects.

## Supplemental Material

(1.3 MB) PDF
