# Supplementary material for: Particulate Air Pollution Exposure and Expression of Viral and Human MicroRNAs in Blood: The Beijing Truck Driver Air Pollution Study
Source: Environ Health Perspect. 2015 Jun 12;124(3):344–50. doi: 10.1289/ehp.1408519 (PMC4786978; doi:10.1289/ehp.1408519)
Supplement: (1.3 MB) PDF [file ehp.1408519.s001.acco.pdf]

Environ Health Perspect
DOI: 10.1289/ehp.1408519

Note to Readers: *EHP* strives to ensure that all journal content is accessible to all readers. However, some figures and Supplemental Material published in *EHP* articles may not conform to 508 standards due to the complexity of the information being presented. If you need assistance accessing journal content, please contact ehp508@niehs.nih.gov. Our staff will work with you to assess and meet your accessibility needs within 3 working days.

Supplemental Material

Particulate Air Pollution Exposure and Expression of Viral and Human MicroRNAs in Blood: The Beijing Truck Driver Air Pollution Study

Lifang Hou, Jitendra Barupal, Wei Zhang, Yinan Zheng, Lei Liu, Xiao Zhang, Chang Dou, John P. McCracken, Anaité Díaz, Valeria Motta, Marco Sanchez-Guerra, Katherine Rose Wolf, Pier Alberto Bertazzi, Joel D. Schwartz, Sheng Wang, and Andrea A. Baccarelli

Table of Contents

Figure S1. Enrichment factors (EFs) of the elemental components of $PM_{2.5}$ in truck drivers (TDs) relative to office workers (OW). EFs were computed as follows: EF(x)t= $[x_{(TD)}/PM_{2.5(TD)}]/[x_{(OW)}/PM_{2.5(OW)}]$; where $[x_{(TD)}/ PM_{2.5(TD)}]$ is the ratio of the mean concentration of element x over the mean $PM_{2.5}$ mass in truck drivers; and $[x_{(OW)}/PM_{2.5(OW)}]$ is the ratio of the mean concentration of the same element x over the mean $PM_{2.5}$ mass in office workers. EF >1 indicates the elemental component concentrations in truck driver exceed those expected in office workers.

Figure S2. Cross-platform correlation across 20 randomly selected independent samples. Confirmation of the nCounter miRNA data was evaluated in 20 randomly selected samples using the TaqMan OpenArray microRNA expression profiling with the QuantStudio 12K Flex Real-Time PCR System. The two independent platforms demonstrated comparable miRNA profiles in these random samples.

Figure S3. Overview of the bioinformatics and biostatistics analysis. The raw miRNA expression data from 228 samples was processed using the NanoStringNorm R package. Target mRNAs of miRNAs associated with short-term changes in EC exposure were selected from three

databases (miRTarBase, TarBase, and miRBase). For genes selection in miRBase, two cutoffs, 10^{-6} and 10^{-8}, were chosen. Genes selected in miRBase with a cutoff of 10^{-8} along with genes from experimental databases were used for Reactome functional networks analyses. FDR, false discovery rate.

Figure S4. Overview of the sample relations based on the PCA plot from 240 observations. A total of 12 observations (all were different participants) in the elongated circle were considered to be obvious outliers based on the PCA plot. NanoString plate numbers were labeled for these 12 samples.

Figure S5. Technical robustness of NanoString plates. Plate numbers are shown on the X-axis. Expression values of positive controls (log_2) are shown on the Y-axis. Each bar represents a plate. Plates were technically robust (*P*-value = 0.9951, one-way ANOVA).

Figure S6. Plate effects on miRNA expression based on PCA plot using pre-processed data. Numbers in the plot represent plate numbers. Negligible plate effects visually observed. Though samples were not randomized by examination day, distinct pattern of miRNA profiles due to plate design were not observed.

Figure S7. Venn diagram showing number of enriched pathways by KEGG and GO (Biological Process) analysis. miRBase assigns *P*-values to individual miRNA-3'-UTR target binding site, so different set of genes can be predicated with a miRBase threshold. Circle color represents miRBase threshold (10^{-6} in blue and 10^{-8} in green). KEGG analysis shows that in the pooled analysis of both groups, only one pathway was found at both thresholds (A). In office workers, most of the pathways (17) were found at both thresholds (B). In truck drivers, 17 out of 19 total pathways were found at both thresholds (C). GO analysis shows that no GO term was robust in both group analyses (D). In office workers, 23 of the 31 total GO terms were found at both thresholds. In truck drivers, 13 of 20 total GO terms were found at both thresholds (F).

Figure S8. Functional analysis using Gene Ontology (GO) biological process terms. GO analysis was performed using combined target genes of all miRNAs with significant changes in each analysis group. Gene sets containing a minimum of eight genes at FDR<1% were considered significant. miRBase assigns *P*-values to individual miRNA-3'-UTR target binding site, so different set of genes can be predicated with a miRBase threshold. Two threshold cutoffs (i.e. 10^{-6} and 10^{-8}) in miRBase database are shown on X-axis. Color of a tile represents enrichment of a GO term; Green= YES, Red= NO. GO term classification which is based on title of a GO term were shown on the right side. In the pooled analysis of both groups, 3 out of 5 total biological processes were related to biological regulation. In office workers, more than 35% of biological processes were related to immune response, and in truck drivers, 50% of biological processes were related to cell proliferation and differentiation. Two biological processes were common in all analysis groups. Seven biological processes were common between office workers and truck drivers.

Table S1. Percent change in miRNA expression level with one μg/m^3 increase in Elemental Carbon (EC) at FDR <20%.

Table S2. Significance of interaction between the exposure (short term exposure to EC) and occupational groups for miRNAs associated with EC by group analysis at FDR<10%.

Table S3. Percent change in miRNA expression level with one-unit increase in elemental carbon (EC) (non-smokers only).

Table S4. Change in miRNA expression level (log2) with one unit increase in PM_{10}.

Table S5. Percent change in miRNA expression level with one-unit increase in $PM_{2.5}$.

Table S6. KEGG pathway enrichment analysis for targets of significant EC-associated miRNAs (for miRBase gene targets at *P*-value <10^{-8}).

Table S7. Gene Ontology (biological term) enrichment analysis (FDR< 1%) for significant EC-associated miRNAs (miRBase gene targets at *P*-value <10^{-8}).

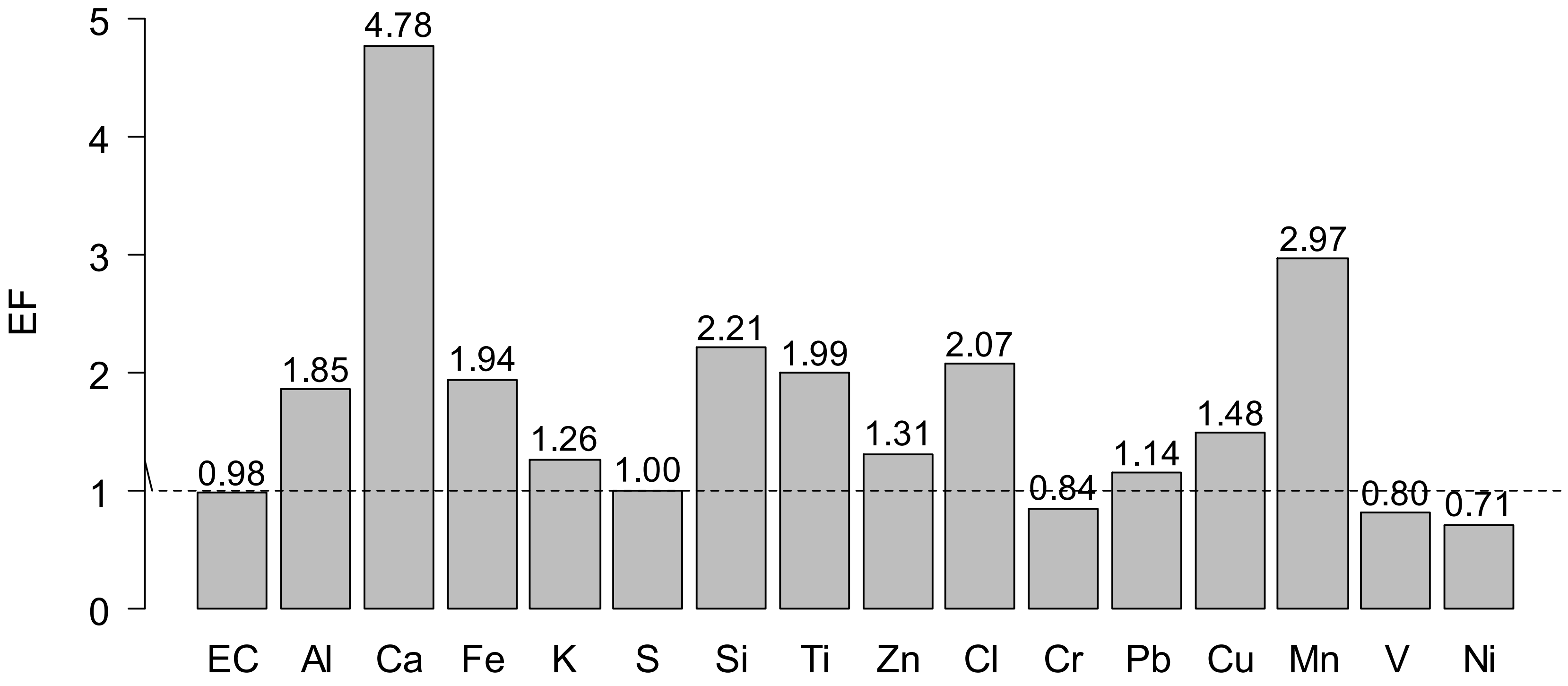

Figure S1. Enrichment factors (EFs) of the elemental components of $PM_{2.5}$ in truck drivers (TDs) relative to office workers (OW). EFs were computed as follows: EF(x)t= $[x_{(TD)}/PM_{2.5(TD)}]/[x_{(OW)}/PM_{2.5(OW)}]$; where $[x_{(TD)}/ PM_{2.5(TD)}]$ is the ratio of the mean concentration of element x over the mean $PM_{2.5}$ mass in truck drivers; and $[x_{(OW)}/PM_{2.5(OW)}]$ is the ratio of the mean concentration of the same element x over the mean $PM_{2.5}$ mass in office workers. EF >1 indicates the elemental component concentrations in truck driver exceed those expected in office workers.

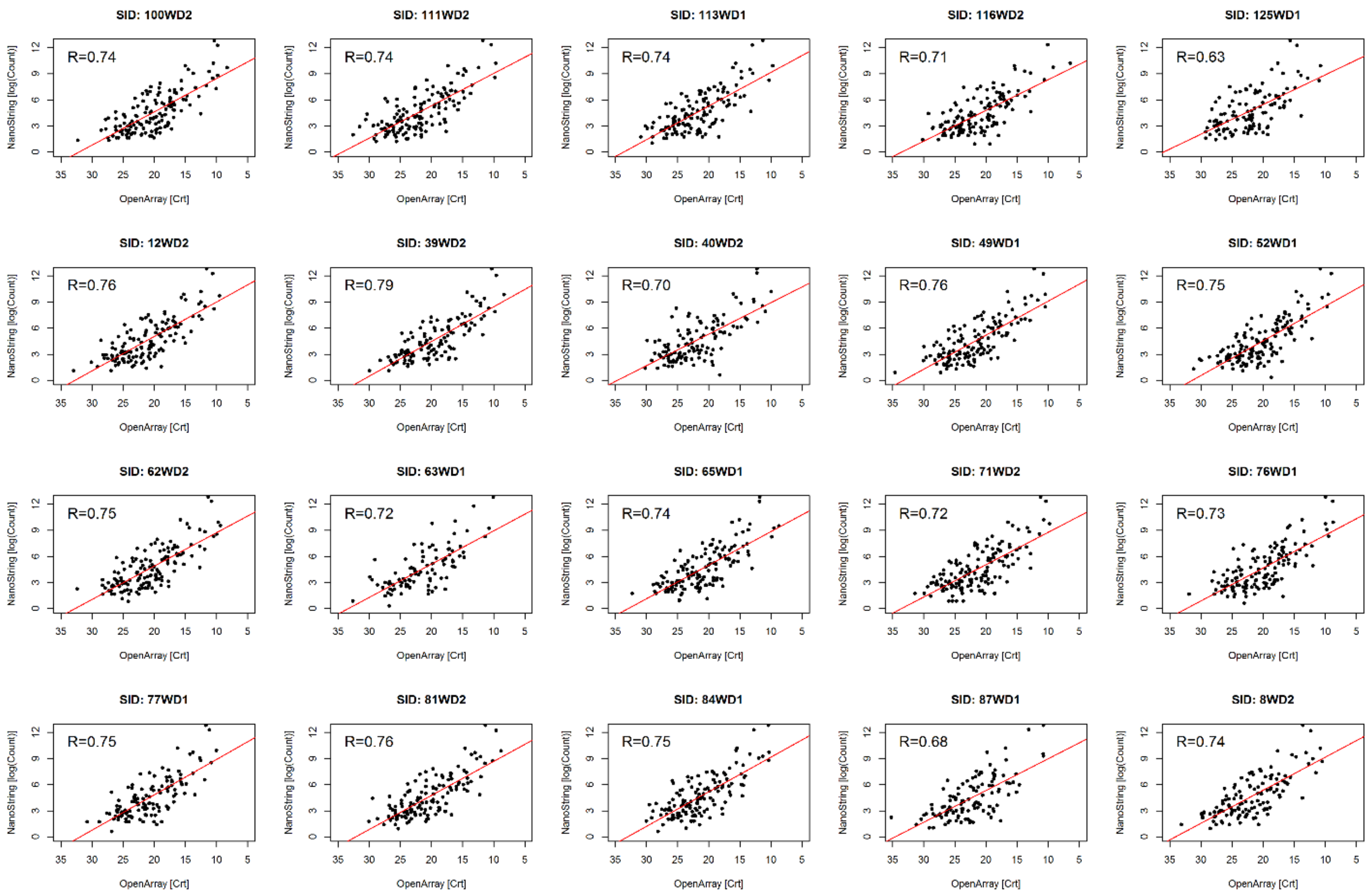

Figure S2. Cross-platform correlation across 20 randomly selected independent samples. Confirmation of the nCounter miRNA data was evaluated in 20 randomly selected samples using the TaqMan OpenArray microRNA expression profiling with the QuantStudio 12K Flex Real-Time PCR System. The two independent platforms demonstrated comparable miRNA profiles in these random samples.

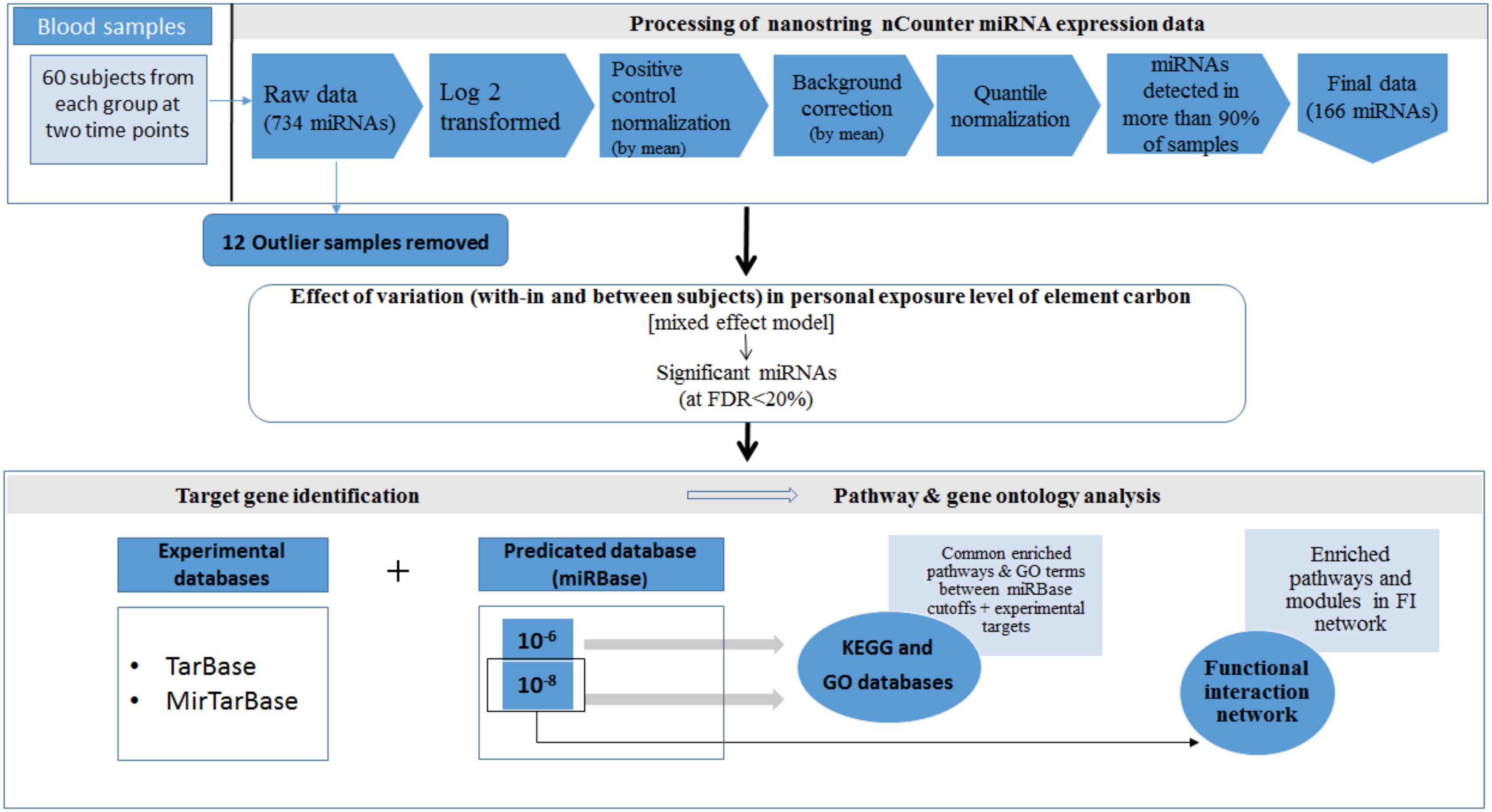

Figure S3. Overview of the bioinformatics and biostatistics analysis. The raw miRNA expression data from 228 samples was processed using the NanoStringNorm R package. Target mRNAs of miRNAs associated with short-term changes in EC exposure were selected from three databases (miRTarBase, TarBase, and miRBase). For genes selection in miRBase, two cutoffs, 10^{-6} and 10^{-8}, were chosen. Genes selected in miRBase with a cutoff of 10^{-8} along with genes from experimental databases were used for Reactome functional networks analyses. FDR, false discovery rate;

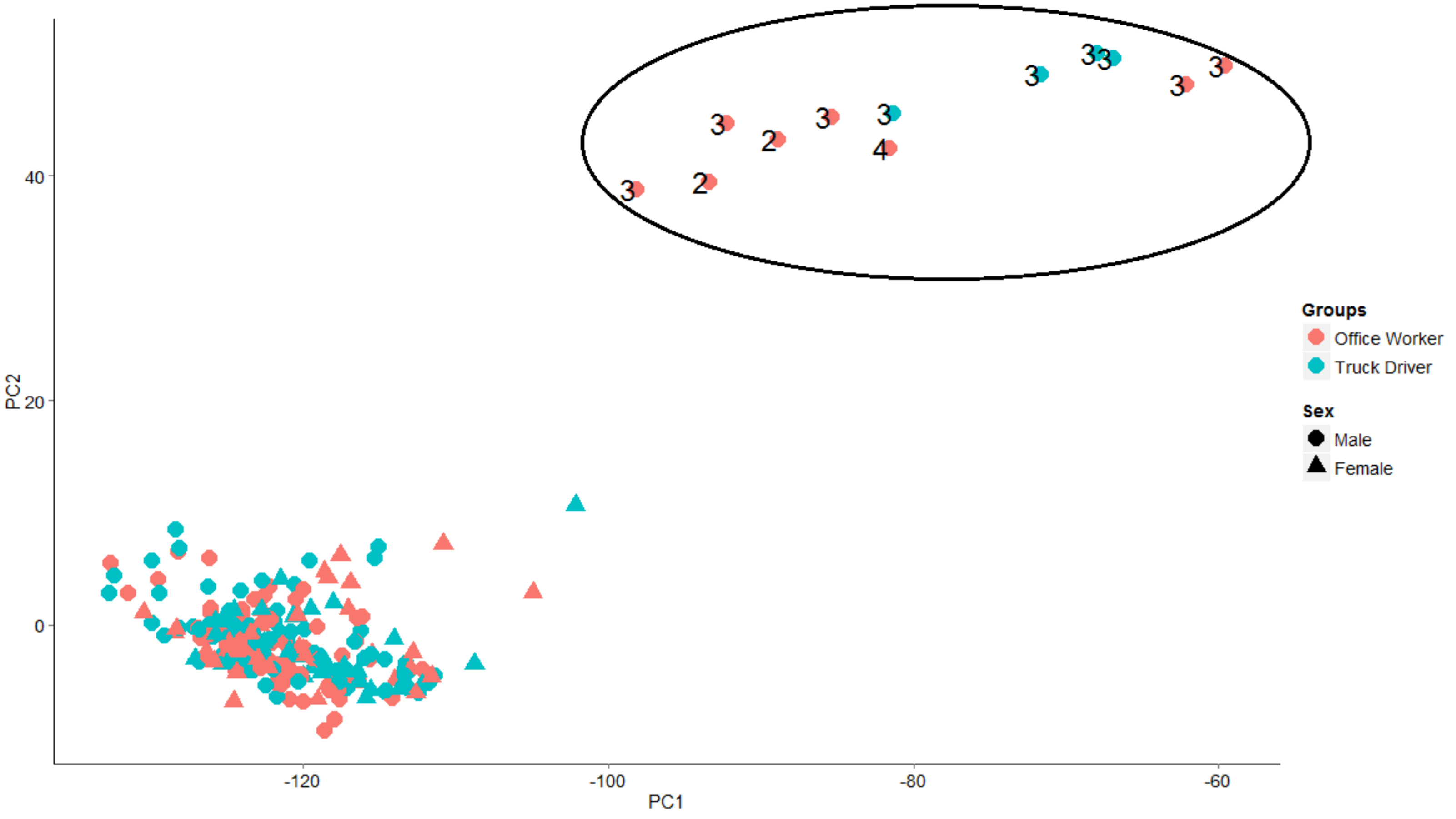

Figure S4. Overview of the sample relations based on the PCA plot from 240 observations. A total of 12 observations (all were different participants) in the elongated circle were considered to be obvious outliers based on the PCA plot. NanoString plate numbers were labeled for these 12 samples.

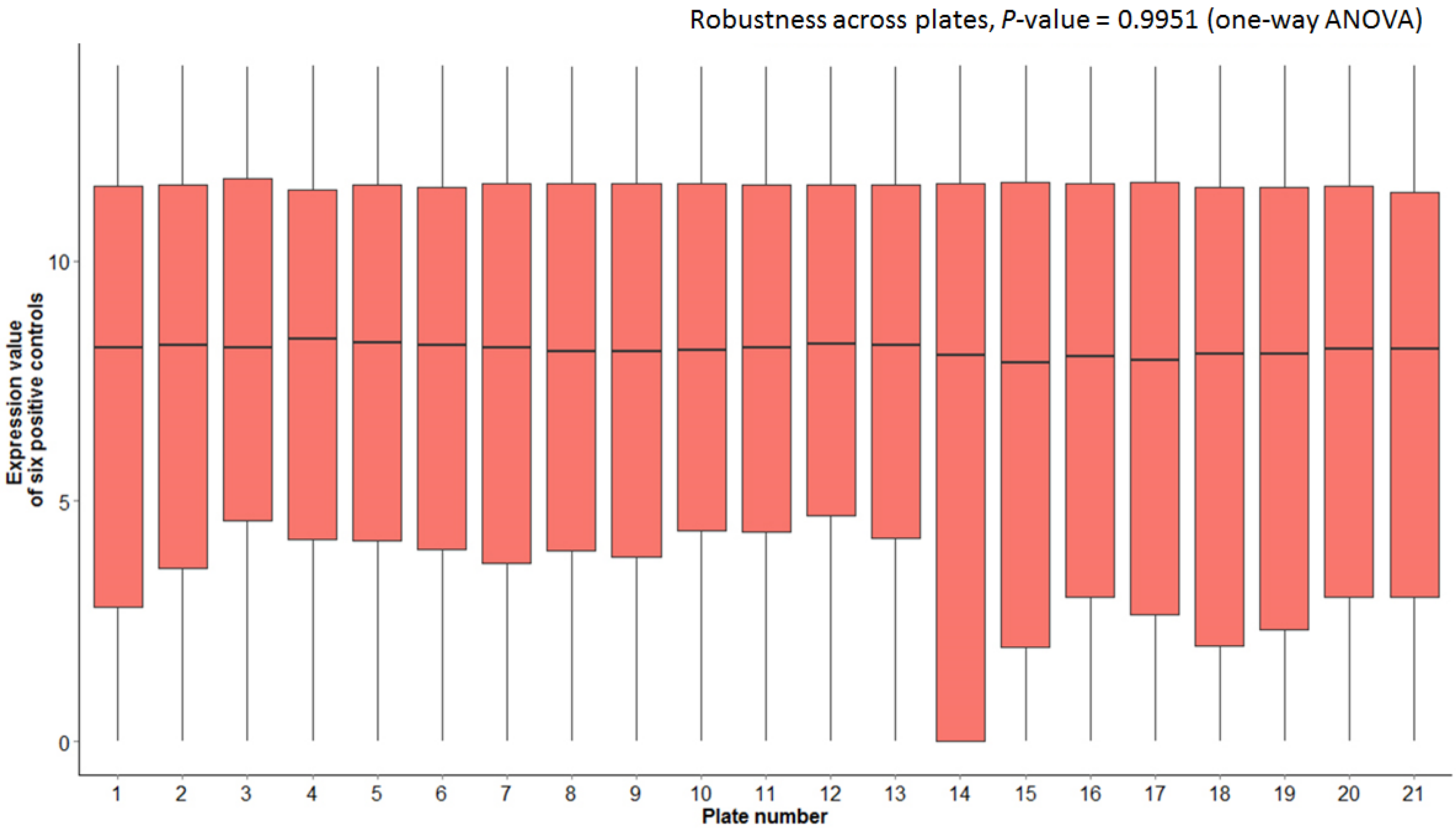

Figure S5. Technical robustness of NanoString plates. Plate numbers are shown on the X-axis. Expression values of positive controls (log_2) are shown on the Y-axis. Each bar represents a plate. Plates were technically robust (*P*-value = 0.9951, one-way ANOVA).

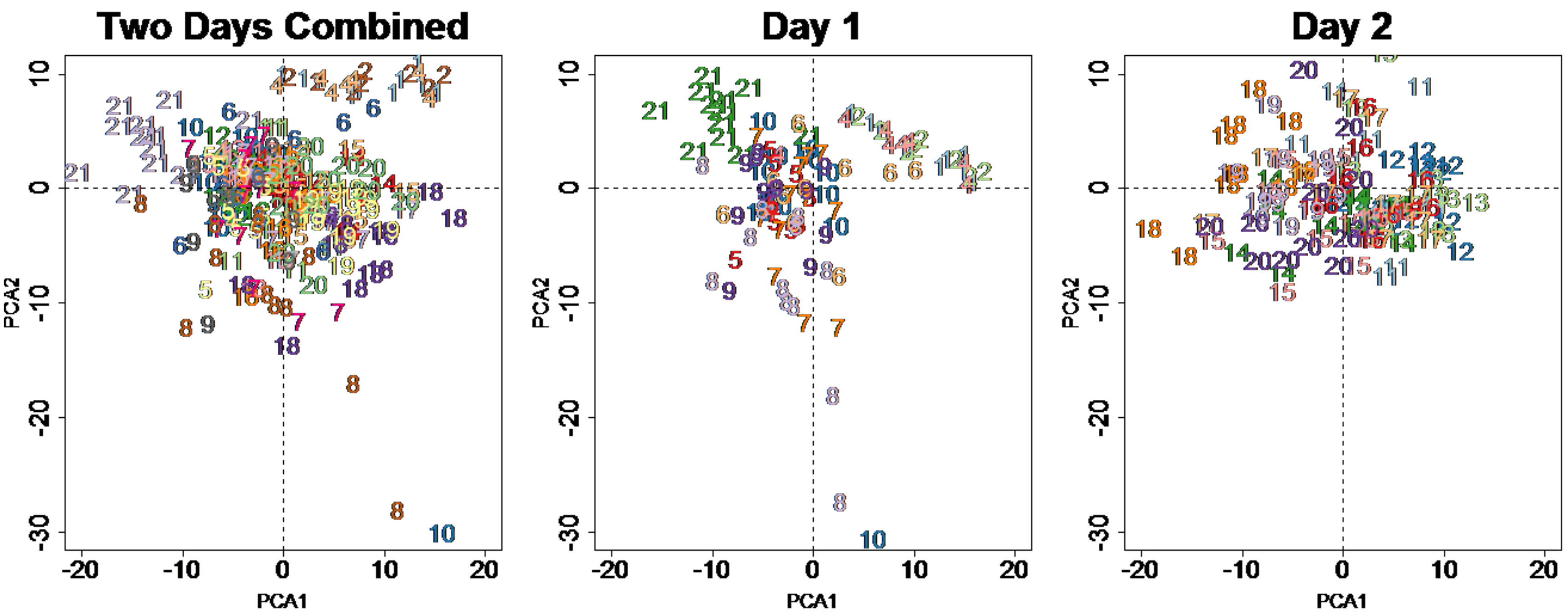

Figure S6. Plate effects on miRNA expression based on PCA plot using pre-processed data. Numbers in the plot represent plate numbers. Negligible plate effects visually observed. Though samples were not randomized by examination day, distinct pattern of miRNA profiles due to plate design were not observed.

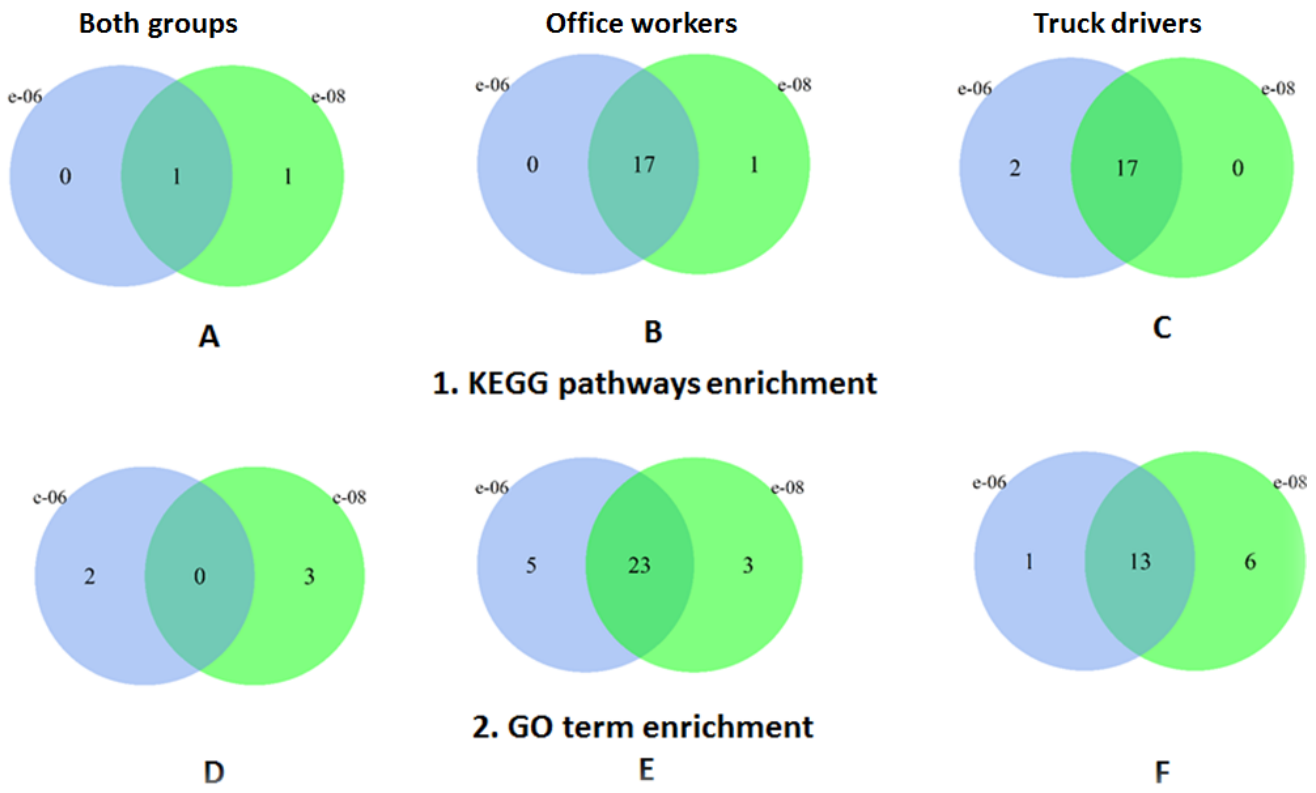

Figure S7. Venn diagram showing number of enriched pathways by KEGG and GO (Biological Process) analysis. miRBase assigns *P*-values to individual miRNA-3'-UTR target binding site, so different set of genes can be predicated with a miRBase threshold. Circle color represents miRBase threshold (10^{-6} in blue and 10^{-8} in green). KEGG analysis shows that in the pooled analysis of both groups, only one pathway was found at both thresholds (A). In office workers, most of the pathways (17) were found at both thresholds (B). In truck drivers, 17 out of 19 total pathways were found at both thresholds (C). GO analysis shows that no GO term was robust in both group analyses (D). In office workers, 23 of the 31 total GO terms were found at both thresholds. In truck drivers, 13 of 20 total GO terms were found at both thresholds (F).

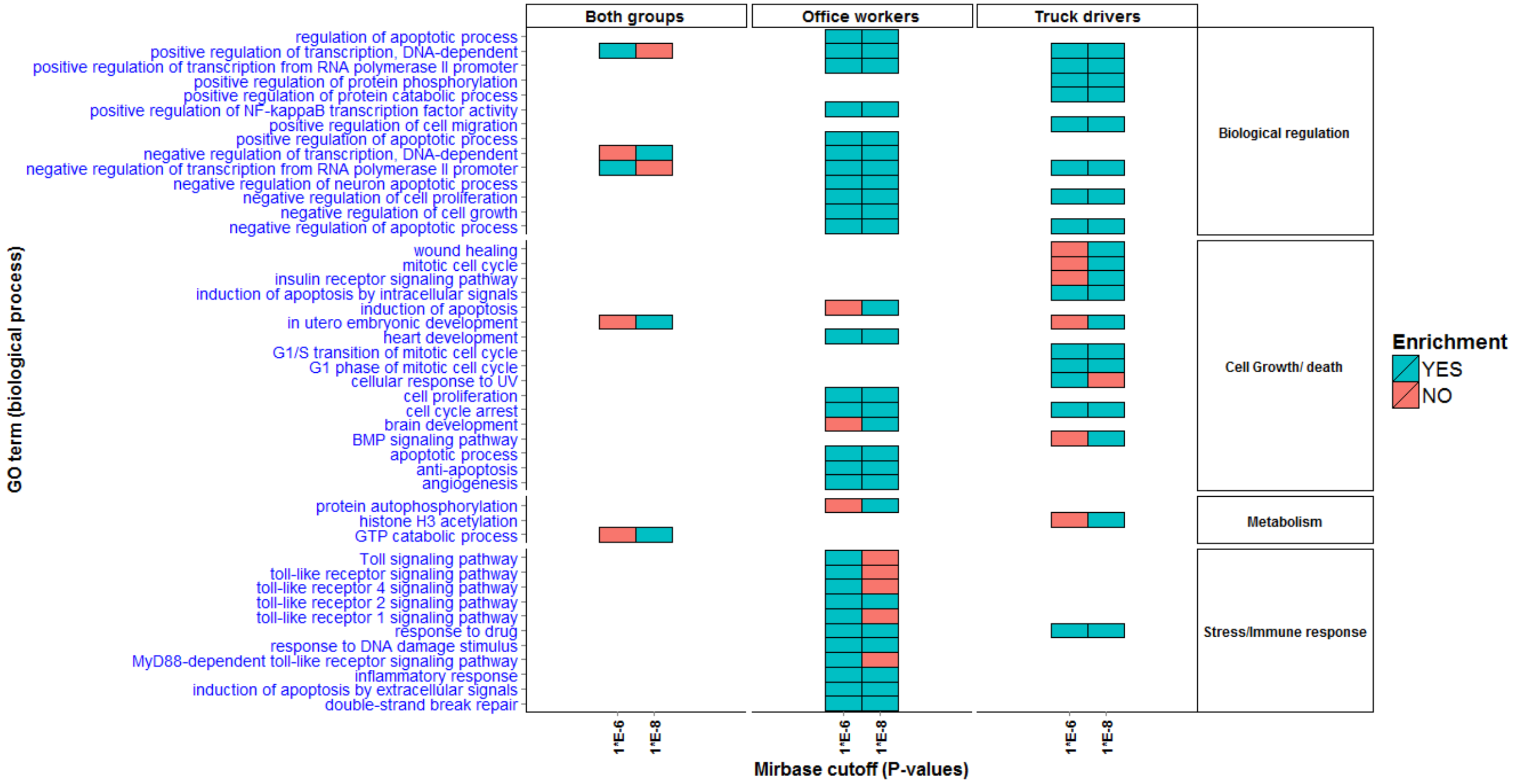

Figure S8. Functional analysis using Gene Ontology (GO) biological process terms. GO analysis was performed using combined target genes of all miRNAs with significant changes in each analysis group. Gene sets containing a minimum of eight genes at FDR<1% were considered significant. miRBase assigns *P*-values to individual miRNA-3'-UTR target binding site, so different set of genes can be predicated with a miRBase threshold. Two threshold cutoffs (i.e. 10^{-6} and 10^{-8}) in miRBase database are shown on X-axis. Color of a tile represents enrichment of a GO term; Green= YES, Red= NO. GO term classification which is based on title of a GO term were shown on the right side. In the pooled analysis of both groups, 3 out of 5 total biological processes were related to biological regulation. In office workers, more than 35% of biological processes were related to immune response, and in truck drivers, 50% of biological processes were related to cell proliferation and differentiation. Two biological processes were common in all analysis groups. Seven biological processes were common between office workers and truck drivers.

Table S1. Percent change in miRNA expression level with one µg/m^3 increase in Elemental Carbon (EC) at FDR <20%.

miRNA name	% change (95% CI)	*P*-value	FDR [*]
**Pooled analysis [a] [within subject (short-term) effects]**			
hsa-miR-1537	11.99 (5.07, 9.38)	0.001	0.102
hsa-miR-548h	7.36 (2.69, 2.25)	0.002	0.145
hsa-miR-200a	1.41 (0.43, 0.40)	0.005	0.145
hsa-miR-520a-3p	11.13 (3.21, 9.66)	0.006	0.145
hsa-miR-155	4.79 (1.25, 0.46)	0.008	0.145
hsa-miR-501-3p	8.43 (2.04, 5.21)	0.010	0.145
hsa-miR-647	7.33 (1.23, 3.70)	0.019	0.193
**Office workers [b] [within subject (short-term) effects]**			
ebv-miR-BART2-5p	7.08 (3.04, 11.28)	0.001	0.062
hsa-miR-548h	16.97 (6.86, 28.03)	0.001	0.062
hsa-miR-1243	13.67 (4.95, 23.13)	0.002	0.062
hsa-miR-181b/181d	12.12 (4.29, 20.54)	0.003	0.062
hsa-miR-302f	7.96 (2.65, 13.54)	0.004	0.062
hsa-miR-647	19.03 (6.10, 33.53)	0.004	0.062
hsa-miR-340	9.44 (3.17, 16.08)	0.004	0.062
hsa-miR-210	-7.92 (-12.85, -2.71)	0.004	0.062
hsa-miR-532-3p	-1.55 (-2.58, -0.52)	0.004	0.062
hsa-miR-642	7.62 (2.43, 13.08)	0.004	0.062
ebv-miR-BART6-5p	-24.19 (-37.17, -8.53)	0.005	0.062
hsa-miR-423-3p	-2.32 (-3.88, -0.72)	0.005	0.062
hsa-miR-484	-4.19 (-6.99, -1.31)	0.006	0.062
hsa-miR-937	2.14 (0.68, 3.62)	0.006	0.062
hsa-miR-125a-5p	-2.84 (-4.81, -0.82)	0.008	0.084
hsa-miR-21	4.46 (1.10, 7.94)	0.011	0.108
hsa-miR-154	2.63 (0.65, 4.66)	0.012	0.108
hsa-miR-10a	-17.23 (-28.65, -4.00)	0.014	0.120
hsa-miR-142-3p	3.80 (0.78, 6.91)	0.015	0.120
hsa-miR-600	-15.97 (-26.80, -3.55)	0.015	0.120

miRNA name	% change (95% CI)	*P*-value	FDR [*]
hsa-miR-146a	2.67 (0.52, 4.87)	0.018	0.129
hsa-miR-2114	19.60 (3.60, 38.07)	0.018	0.129
hsa-miR-150	-4.04 (-7.27, -0.70)	0.021	0.142
hsa-miR-1283	-13.65 (-23.94, -1.97)	0.026	0.170
hsa-miR-1274a	-8.39 (-15.20, -1.03)	0.029	0.172
hsa-miR-328	-11.70 (-21.04, -1.25)	0.032	0.172
kshv-miR-K12-9	13.40 (1.25, 27.00)	0.032	0.172
hsa-miR-515-5p	16.19 (1.13, 33.50)	0.037	0.185
**Truck drivers [b] [within subject (short-term) effects]**			
hsa-miR-513a-3p	1.49 (0.60, 2.39)	0.002	0.064
hsa-miR-1537	14.03 (5.17, 23.63)	0.002	0.064
hsa-miR-155	6.73 (2.51, 11.13)	0.002	0.064
ebv-miR-BHRF1-3	0.98 (0.38, 1.59)	0.002	0.064
ebv-miR-BHRF1-1	0.98 (0.36, 1.59)	0.002	0.064
hsa-miR-520a-3p	14.64 (4.92, 25.26)	0.003	0.064
hsa-miR-125a-5p	3.15 (1.08, 5.26)	0.004	0.064
ebv-miR-BART9	1.59 (0.54, 2.66)	0.004	0.064
hsa-miR-652	13.62 (4.30, 23.77)	0.004	0.064
hsa-miR-501-3p	12.70 (3.96, 22.17)	0.005	0.064
hsa-let-7b	1.76 (0.57, 2.97)	0.005	0.064
hsa-miR-320a	1.55 (0.48, 2.63)	0.005	0.064
hiv1-miR-H1	1.30 (0.41, 2.20)	0.006	0.066
hsa-miR-518f	6.73 (1.85, 11.83)	0.008	0.068
hsa-miR-208b	7.10 (1.91, 12.54)	0.009	0.070
hsa-miR-192	-11.69 (-19.82, -2.73)	0.013	0.080
hsa-miR-361-3p	2.55 (0.55, 4.59)	0.014	0.080
hsa-miR-1308	7.26 (1.50, 13.34)	0.014	0.080
hsa-miR-125b	2.42 (0.52, 4.36)	0.016	0.083
hsa-miR-1979	2.00 (0.39, 3.63)	0.016	0.083
hsa-miR-1274a	6.15 (1.14, 11.41)	0.017	0.083
hsa-miR-96	2.38 (0.43, 4.38)	0.018	0.083

miRNA name	% change (95% CI)	*P*-value	FDR [*]
hsa-miR-195	6.25 (1.02, 11.76)	0.021	0.090
hsa-miR-10b	9.39 (1.38, 18.04)	0.023	0.097
hsa-miR-206	4.20 (0.47, 8.06)	0.030	0.110
hsa-miR-600	10.13 (0.85, 20.26)	0.034	0.118
hsa-miR-1283	8.98 (0.75, 17.89)	0.034	0.118
hsa-miR-10a	9.62 (0.76, 19.25)	0.035	0.118
hsa-miR-708	10.49 (0.78, 21.13)	0.036	0.118

[a]Adjusted for occupational group, age, sex, BMI, smoking status, number of cigarettes smoked, work hours, examination date, and humidity and temperature of the study days.

[b]Adjusted for age, sex, BMI, smoking status, number of cigarettes smoked, work hours, examination date, and humidity and temperature of the study days.

[*] FDRs (Benjamini-Hochberg false discovery rates) <20% were considered significant. Non-significant results are not shown.

Table S2. Significance of interaction between the exposure (short term exposure to EC) and occupational groups for miRNAs associated with EC by group analysis at FDR<10%.

miRNA	*P*-value *
**Significant miRNAs in office workers**	
ebv-miR-BART6-5p	<0.001
hsa-miR-532-3p	<0.001
hsa-miR-423-3p	0.001
hsa-miR-181b/181d	0.002
hsa-miR-125a-5p	0.002
hsa-miR-1243	0.002
hsa-miR-484	0.005
hsa-miR-647	0.006
hsa-miR-302f	0.022
hsa-miR-548h	0.024
hsa-miR-340	0.027
hsa-miR-642	0.033
ebv-miR-BART2-5p	0.034
hsa-miR-937	0.036
hsa-miR-210	0.247
**Significant miRNAs in truck drivers**	
hsa-miR-1274a	0.001
hsa-miR-518f	0.002
hsa-miR-125a-5p	0.002
hsa-miR-320a	0.002
hsa-miR-652	0.003
hsa-miR-192	0.003
hsa-miR-96	0.004
hsa-miR-520a-3p	0.007
hsa-miR-501-3p	0.007
hsa-miR-1979	0.009
hsa-let-7b	0.011

miRNA	*P*-value *
hsa-miR-1308	0.012
hsa-miR-361-3p	0.013
ebv-miR-BHRF1-1	0.015
ebv-miR-BHRF1-3	0.020
hsa-miR-208b	0.029
hsa-miR-10b	0.037
hsa-miR-513a-3p	0.039
hiv1-miR-H1	0.048
hsa-miR-195	0.084
hsa-miR-155	0.106
hsa-miR-125b	0.174
ebv-miR-BART9	0.175
hsa-miR-1537	0.630

* *P*-value for interaction between change in EC level and occupational group (office worker or truck driver). The model adjusted for group, age, sex, BMI, smoking status, number of cigarettes smoked, work hours, examination date, and humidity and temperature of the study days.

Table S3. Percent change in miRNA expression level with one-unit increase in elemental carbon (EC) (non-smokers only). [a]

miRNA name	% change (95% CI)	*P*-value	FDR [*]
**Office workers**			
hsa-miR-451	1.67 (0.78, 2.57)	0.001	0.123
ebv-miR-BART2-5p [b]	11.13 (4.22, 18.49)	0.003	0.128
hsa-miR-92a	-1.59 (-2.56, -0.61)	0.003	0.128
hsa-miR-642 [b]	13.26 (4.40, 22.88)	0.004	0.128
hsa-miR-340 [b]	16.75 (5.58, 29.10)	0.005	0.128
hsa-miR-548h [b,d]	21.16 (6.68, 37.61)	0.005	0.128
hsa-miR-937 [b]	3.48 (1.11, 5.89)	0.006	0.128
ebv-miR-BART6-5p [b]	-28.26 (-43.19, -9.40)	0.007	0.128
hsa-miR-21[b]	7.93 (2.37, 13.79)	0.008	0.128
hsa-miR-1243 [b]	21.66 (5.96, 39.68)	0.008	0.128
hsa-miR-154 [b]	4.52 (1.32, 7.82)	0.009	0.128
hsa-miR-302f [b]	12.18 (3.31, 21.81)	0.010	0.131
**Truck drivers**			
hsa-miR-374b	-11.00 (-16.70, -4.91)	0.001	0.180
hsa-miR-15b	4.59 (1.73, 7.53)	0.003	0.180
hsa-miR-183	3.15 (1.13, 5.22)	0.004	0.180
ebv-miR-BART9 [c]	1.79 (0.59, 3.00)	0.005	0.180
ebv-miR-BHRF1-1 [c]	1.32 (0.43, 2.22)	0.005	0.180

[a] Adjusted for age, sex, BMI, smoking status, number of cigarettes smoked, work hours, examination date, and humidity and temperature of the study days.

[b] miRNA significantly associated with EC in analysis that included all participants of office workers.

[c] miRNA significantly associated with EC in analysis that included all participants of truck drivers.

[d] miRNA significantly associated with EC in analysis that included all participants.

[*] FDRs (Benjamini-Hochberg false discovery rates) <20% were considered significant results. Non-significant results are not shown.

Table S4. Change in miRNA expression level (log2) with one unit increase in PM_{10}.

miRNA name	% change (95% CI)	*P*-value	FDR *
**Pooled analysis [a] [Within Subject (short-term) effects]**			
hsa-miR-515-5p	0.99 (0.41, 1.57)	0.001	0.133
**Office workers [b] [Within subject (short-term) effects]**			
hsa-miR-10b [d]	-1.47 (-2.02, -0.92)	<0.001	<0.001
hsa-miR-648	-1.55 (-2.16, -0.94)	<0.001	<0.001
hsa-miR-137	-1.68 (-2.44, -0.91)	<0.001	0.004
hsa-miR-10a [c,d]	-2.02 (-2.95, -1.08)	<0.001	0.004
hsa-miR-1183	-1.08 (-1.61, -0.54)	<0.001	0.004
hsa-miR-501-3p [d,e]	-1.21 (-1.81, -0.61)	<0.001	0.004
ebv-miR-BART6-5p [d]	-2.05 (-3.27, -0.82)	0.002	0.012
hsa-miR-300	-1.51 (-2.44, -0.56)	0.002	0.014
hsa-miR-559	-1.49 (-2.43, -0.55)	0.003	0.016
hsa-miR-515-5p [c]	1.39 (0.48, 2.31)	0.004	0.017
hsa-miR-651[c]	-1.48 (-2.44, -0.52)	0.004	0.019
hsa-miR-520a-3p [d,e]	-1.39 (-2.36, -0.41)	0.008	0.030

[a] Adjusted for occupational group, age, sex, BMI, smoking status, number of cigarettes smoked, work hours, examination date, and humidity and temperature of the study days.

[b] Adjusted for age, sex, BMI, smoking status, number of cigarettes smoked, work hours, examination date, and humidity and temperature of the study days.

[c] miRNA significantly associated with EC in office workers.

[d] miRNA significantly associated with EC in truck drivers.

[e] miRNA significantly associated with EC in pooled analysis.

[*] FDRs (Benjamini-Hochberg false discovery rates) <20% were considered significant. Non-significant results are not shown.

Table S5. Percent change in miRNA expression level with one-unit increase in $PM_{2.5}$.

miRNA name	% change (95% CI)	*P*-value [*]	FDR
**Pooled analysis [a] [Between-subjects effects]**			
hsa-miR-1537	5.86 (1.51, 10.40)	0.008	0.982
**Pooled analysis [a] [Within subject (short-term) effects]**			
hsa-miR-1537	6.35 (1.87, 11.03)	0.006	0.886
hsa-miR-374b	-3.02 (-5.37, -0.61)	0.015	0.886
**Office workers [b] [Between-subjects effects]**			
hsa-miR-647	12.10 (4.58, 20.16)	0.002	0.287
hsa-miR-181b/181d	6.25 (1.65, 11.06)	0.009	0.468
hsa-miR-302f	4.00 (0.84, 7.26)	0.014	0.468
hsa-miR-601	9.58 (1.92, 17.82)	0.015	0.468
ebv-miR-BART6-5p	-12.78 (-22.11, -2.34)	0.020	0.468
hsa-miR-1243	5.93 (0.85, 11.27)	0.024	0.492
hsa-miR-1323	8.71 (0.67, 17.40)	0.036	0.512
hsa-miR-759	7.14 (0.55, 14.16)	0.036	0.512
**Office workers [b] [Within subject (short-term) effects]**			
hsa-miR-647	11.77 (4.12, 19.98)	0.003	0.451
hsa-miR-181b/181d	6.33 (1.63, 11.25)	0.009	0.589
hsa-miR-302f	4.03 (0.81, 7.36)	0.016	0.589
hsa-miR-601	9.72 (1.90, 18.15)	0.016	0.589
hsa-miR-1243	6.04 (0.85, 11.50)	0.024	0.589
hsa-miR-759	7.57 (0.83, 14.77)	0.030	0.589
ebv-miR-BART6-5p	-11.93 (-21.53, -1.15)	0.033	0.589
hsa-miR-647	11.77 (4.12, 19.98)	0.003	0.451
**Truck drivers [b] [Between-subjects effects]**			
hsa-miR-192	-9.74 (-15.62, -3.44)	0.004	0.598
hsa-miR-1537	7.75 (1.75, 14.10)	0.012	0.881
hsa-miR-374b	-3.13 (-5.80, -0.39)	0.028	0.881
**Truck drivers [b] [Within subject (short-term) effects]**			
hsa-miR-192	-10.13 (-16.14, -3.69)	0.003	0.521
hsa-miR-1537	7.56 (1.42, 14.08)	0.017	0.831
hsa-miR-877	-5.54 (-10.02, -0.84)	0.024	0.831

[a] Adjusted for age, sex, BMI, smoking status, group, number of cigarettes smoked, work hours, examination date, and humidity and temperature of the study days.

[b] Adjusted for age, sex, BMI, smoking status, number of cigarettes smoked, work hours, examination date, and humidity and temperature of the study days.

[*] *P*-values <0.05 are included.

Table S6. KEGG pathway enrichment analysis for targets of significant EC-associated miRNAs (for miRBase gene targets at *P*-value $<10^{-8}$).

KEGG ID	Pathway	Gene list [a]	Total no. of genes [b]	*P*-value [*]	FDR [**]	Enrichment score	KEGG Higher Categories (Level-1)	KEGG Higher Categories (Level-2)
**Pooled analysis**								
hsa05210	Colorectal cancer	8	62	<0.001	0.001	9.92	Human Disease	Cancer
hsa05200	Pathways in cancer	13	326	0.001	0.028	3.07	Human Disease	Cancer
**Office workers**								
hsa05200	Pathways in cancer	24	326	<0.001	<0.001	4.53	Human Disease	Cancer
hsa05219	Bladder cancer	10	42	<0.001	<0.001	14.65	Human Disease	Cancer
hsa05212	Pancreatic cancer	11	70	<0.001	<0.001	9.67	Human Disease	Cancer
hsa05222	Small cell lung cancer	10	85	<0.001	<0.001	7.24	Human Disease	Cancer
hsa05215	Prostate cancer	10	89	<0.001	<0.001	6.91	Human Disease	Cancer
hsa04620	Toll-like receptor signaling pathway	10	102	<0.001	<0.001	6.03	Organismal Systems	Immune system
hsa05142	Chagas disease (American trypanosomiasis)	10	104	<0.001	<0.001	5.91	Human Disease	Infectious diseases: Parasitic
hsa05210	Colorectal cancer	8	62	<0.001	<0.001	7.94	Human Disease	Cancer
hsa04210	Apoptosis	9	87	<0.001	<0.001	6.36	Cellular Processes	Cell growth and death
hsa05218	Melanoma	8	71	<0.001	0.001	6.93	Human Disease	Cancer
hsa04520	Adherens junction	8	73	<0.001	0.001	6.74	Cellular Processes	Cell Communication
hsa05220	Chronic myeloid leukemia	8	73	<0.001	0.001	6.74	Human Disease	Cancer
hsa04060	Cytokine-cytokine receptor interaction	15	265	<0.001	0.001	3.48	Environmental Information Processing	Signaling molecules and interaction
hsa04010	MAPK signaling pathway	15	268	<0.001	0.001	3.44	Environmental Information Processing	Signal transduction
hsa04722	Neurotrophin signaling pathway	9	127	<0.001	0.003	4.36	Organismal Systems	Nervous system

KEGG ID	Pathway	Gene list [a]	Total no. of genes [b]	*P*-value [*]	FDR [**]	Enrichment score	KEGG Higher Categories (Level-1)	KEGG Higher Categories (Level-2)
hsa04110	Cell cycle	8	124	0.002	0.008	3.97	Cellular Processes	Cell growth and death
hsa05145	Toxoplasmosis	8	132	0.002	0.011	3.73	Human Diseases	Infectious diseases: Parasitic
hsa04062	Chemokine signaling pathway	9	189	0.006	0.024	2.93	Organismal Systems	Immune system
**Truck drivers**								
hsa05200	Pathways in cancer	35	326	<0.001	<0.001	2.99	Human Disease	Cancer
hsa05210	Colorectal cancer	13	62	<0.001	<0.001	5.84	Human Disease	Cancer
hsa05218	Melanoma	14	71	<0.001	<0.001	5.49	Human Disease	Cancer
hsa05219	Bladder cancer	11	42	<0.001	<0.001	7.29	Human Disease	Cancer
hsa05215	Prostate cancer	15	89	<0.001	<0.001	4.69	Human Disease	Cancer
hsa04110	Cell cycle	17	124	<0.001	<0.001	3.82	Cellular Processes	Cell growth and death
hsa05213	Endometrial cancer	11	52	<0.001	<0.001	5.89	Human Disease	Cancer
hsa05223	Non-small cell lung cancer	11	54	<0.001	<0.001	5.67	Human Disease	Cancer
hsa05212	Pancreatic cancer	12	70	<0.001	0.001	4.77	Human Disease	Cancer
hsa05214	Glioma	11	65	<0.001	0.001	4.71	Human Disease	Cancer
hsa05221	Acute myeloid leukemia	10	57	<0.001	0.002	4.88	Human Disease	Cancer
hsa05220	Chronic myeloid leukemia	11	73	<0.001	0.002	4.19	Human Disease	Cancer
hsa04115	p53 signaling pathway	10	68	<0.001	0.005	4.09	Cellular Processes	Cell growth and death
hsa00240	Pyrimidine metabolism	12	99	0.001	0.007	3.37	Metabolism	Nucleotide metabolism
hsa00230	Purine metabolism	15	162	0.002	0.017	2.58	Metabolism	Nucleotide metabolism
hsa04120	Ubiquitin mediated proteolysis	13	135	0.002	0.022	2.68	Genetic Information Processing	Folding
hsa04910	Insulin signaling pathway	13	138	0.003	0.025	2.62	Organismal Systems	Endocrine system

[a] No. of genes in a pathway (from 76 Entrez gene IDs in all participants combined, 95 Entrez gene IDs in office workers and 210 Entrez gene IDs in truck drivers, for genes selected at miRBase 10^{-8}).

[b] Number of genes in the a pathway (from 5,844 total genes as background).

[*] *P*-values calculated using Fisher's exact test.

[**] FDRs (Benjamini-Hochberg false discovery rates) <5% were considered significant. Non-significant results are not shown.

Table S7. Gene Ontology (biological term) enrichment analysis (FDR< 1%) for significant EC-associated miRNAs (miRBase gene targets at *P*-value $<10^{-8}$).

GO ID	Biological process term	Gene list [a]	Total no. of genes [b]	*P*-value [*]	FDR [**]	Enrichment score	GO term categories [c]
**Pooled analysis**							
GO:0001701	in utero embryonic development	10	165	<0.001	0.006	5.44	Cell Growth/ death
GO:0006184	GTP catabolic process	10	178	<0.001	0.008	5.04	Metabolism
GO:0045892	negative regulation of transcription, DNA-dependent	16	440	<0.001	0.009	3.26	Biological regulation
**Office workers**							
GO:0006915	apoptotic process	36	702	<0.001	<0.001	4.60	Cell Growth/ death
GO:0043066	negative regulation of apoptotic process	21	276	<0.001	<0.001	6.83	Biological regulation
GO:0045893	positive regulation of transcription, DNA dependent	28	539	<0.001	<0.001	4.66	Biological regulation
GO:0008285	negative regulation of cell proliferation	22	348	<0.001	<0.001	5.67	Biological regulation
GO:0045944	positive regulation of transcription from RNA polymerase II promoter	29	650	<0.001	<0.001	4.00	Biological regulation
GO:0051092	positive regulation of NF-kappaB transcription factor activity	12	106	<0.001	<0.001	10.16	Biological regulation
GO:0045892	negative regulation of transcription, DNA -dependent	22	440	<0.001	<0.001	4.49	Biological regulation
GO:0042493	response to drug	17	290	<0.001	<0.001	5.26	Stress/Immune response
GO:0000122	negative regulation of transcription from RNA polymerase II promoter	21	454	<0.001	<0.001	4.15	Biological regulation
GO:0007507	heart development	11	133	<0.001	<0.001	7.42	Cell Growth/ Death
GO:0007050	cell cycle arrest	11	137	<0.001	<0.001	7.21	Cell Growth/ Death
GO:0006302	double-strand break repair	8	62	<0.001	<0.001	11.58	Stress/immune response

GO ID	Biological process term	Gene list [a]	Total no. of genes [b]	*P*-value [*]	FDR [**]	Enrichment score	GO term categories [c]
GO:0034134	toll-like receptor 2 signaling pathway	8	72	<0.001	<0.001	9.97	Stress/immune response
GO:0006974	response to DNA damage stimulus	10	130	<0.001	<0.001	6.90	Stress/immune response
GO:0043524	negative regulation of neuron apoptotic process	8	78	<0.001	<0.001	9.21	Biological regulation
GO:0008283	cell proliferation	15	326	<0.001	0.001	4.13	Cell growth/ death
GO:0006916	anti-apoptosis	12	215	<0.001	0.001	5.01	Cell growth/ death
GO:0001525	angiogenesis	11	189	<0.001	0.001	5.22	Cell growth/ death
GO:0006954	inflammatory response	13	273	<0.001	0.001	4.27	Stress/immune response
GO:0008624	induction of apoptosis by extracellular signals	8	110	<0.001	0.002	6.53	Stress/immune response
GO:0043065	positive regulation of apoptotic process	10	181	<0.001	0.002	4.96	Biological regulation
GO:0030308	negative regulation of cell growth	8	118	<0.001	0.003	6.09	Biological regulation
GO:0046777	protein autophosphorylation	9	158	<0.001	0.003	5.11	Metabolism
GO:0042981	regulation of apoptotic process	9	176	<0.001	0.006	4.59	Biological regulation
GO:0006917	induction of apoptosis	9	195	0.001	0.009	4.14	Cell growth/ death
GO:0007420	brain development	8	156	0.001	0.009	4.60	Cell growth/ death
**Truck drivers**							
GO:0008285	negative regulation of cell proliferation	34	348	<0.001	<0.001	3.38	Biological regulation
GO:0000082	G1/S transition of mitotic cell cycle	21	149	<0.001	<0.001	4.87	Cell growth/ death
GO:0043066	negative regulation of apoptotic process	28	276	<0.001	<0.001	3.51	Biological regulation
GO:0045944	positive regulation of transcription from RNA polymerase II promoter	48	650	<0.001	<0.001	2.55	Biological regulation
GO:0045732	positive regulation of protein catabolic process	10	31	<0.001	<0.001	11.15	Metabolism
GO:0045893	positive regulation of transcription	41	539	<0.001	<0.001	2.63	Biological regulation

GO ID	Biological process term	Gene list [a]	Total no. of genes [b]	*P*-value [*]	FDR [**]	Enrichment score	GO term categories [c]
GO:0008629	induction of apoptosis by intracellular signals	11	46	<0.001	<0.001	8.27	Cell growth/ death
GO:0000122	negative regulation of transcription from RNA polymerase II promoter	35	454	<0.001	<0.001	2.67	Biological regulation
GO:0007050	cell cycle arrest	17	137	<0.001	0.001	4.29	Cell growth/death
GO:0042493	response to drug	26	290	<0.001	0.001	3.10	Stress/immune response
GO:0001934	positive regulation of protein phosphorylation	12	75	<0.001	0.001	5.53	Metabolism
GO:0030335	positive regulation of cell migration	14	103	<0.001	0.001	4.70	Cell growth/ death
GO:0000080	G1 phase of mitotic cell cycle	9	40	<0.001	0.001	7.78	Cell growth/death
GO:0030509	BMP signaling pathway	11	70	<0.001	0.003	5.43	Cell growth/death
GO:0000278	mitotic cell cycle	25	324	<0.001	0.004	2.67	Cell growth/death
GO:0042060	wound healing	10	62	<0.001	0.004	5.58	Cell growth/ death
GO:0043966	histone H3 acetylation	8	39	<0.001	0.005	7.09	Metabolism
GO:0001701	in utero embryonic development	16	165	<0.001	0.007	3.35	Cell growth/death
GO:0008286	insulin receptor signaling pathway	15	154	<0.001	0.010	3.37	Cell growth/death

[a] No. of genes in a pathway (from 161 Entrez gene IDs in all participants combined, 161 Entrez gene IDs in office workers, and 418 Entrez gene IDs in truck drivers, for genes selected at miRBase 10^{-8}).

[b] Number of genes in the a pathway (from 14,451 total genes as background).

[c] Based on title of GO term four type of categories were considered; 1. Stress/ immune responses, 2. Biological Regulation, 3. Metabolism 4. Cell growth/death.

[*] *P*-values calculated using Fisher's exact test.

[**] FDRs (Benjamini-Hochberg false discovery rates) <1% were considered significant. Non-significant results are not shown.
